# Nodal Langerhans cell neoplasm: detailing the diagnostic quandaries

**DOI:** 10.4322/acr.2021.344

**Published:** 2021-12-10

**Authors:** Zachariah Chowdhury, Juhi Varshney, Anil Singh, Satvik Khaddar

**Affiliations:** 1 Mahamana Pandit Madanmohan Malaviya Cancer Centre & Homi Bhaba Cancer Hospital, Department of Pathology, Varanasi, Uttar Pradesh, India; 2 Mahamana Pandit Madanmohan Malaviya Cancer Centre & Homi Bhaba Cancer Hospital, Department of Medical Oncology, Varanasi, Uttar Pradesh, India

**Keywords:** histiocytosis, Langerhans cell, lymphoma, pleura, sarcoma

## Abstract

Langerhans cells, found in the supra-basal region of the mucous membranes in the epidermis of the skin, in lymph nodes and thymus, function as antigen-presenting cells within the histiocyte system. Tumors derived from Langerhans cells (LC) can be divided according to the degree of cytological atypia and clinical behavior into Langerhans cell histiocytosis (LCH) and Langerhans cell sarcoma (LCS). LCS is rare, and the nodal presentation is even rarer with challenging histological characteristics. LCS has a dismal overcome despite intensive chemotherapy. Herein, we report a case of a 29-year-old male who presented with generalized lymphadenopathy initially considered as a lymphoma. An outright definitive diagnosis could not be attained in the initial histomorphological and immunohistochemical evaluation, fraught with differential diagnoses. The key to decoding the precise neoplasm was a combination of the cytopathologic features, review of the histomorphology, and extensive immunohistochemical assessment in conjunction with the clinical and positron emission tomography (PET) scan findings. The best diagnosis proffered was a Langerhans cell histiocytosis progressing to Langerhans cell sarcoma. This case highlights the grey zone areas in LC neoplasms, the diagnostic conundrums encountered, the indispensable role of meticulous pathological analysis, and the importance of ancillary studies in hammering out the final diagnosis.

## INTRODUCTION

Langerhans cells (LC) are antigen-presenting cells belonging to the histiocytic system. Histiocyte tumors are amongst the most uncommon neoplasms affecting lymphoid tissues, probably representing less than 1% of all malignancies involving lymph nodes or soft tissues.^1^ Langerhans cell histiocytosis (LCH) and Langerhans cell sarcoma (LCS) constitute proliferative disorders of LCs, which are rarely seen in the daily pathology practice. Differentiating LCS from LCH is challenging because they share identical immunophenotypes, but LCS is an aggressive malignancy with > 50% mortality, and thus, it is important to characterize these lesions accurately.^2^ Most of the LCS reported cases are extranodal, involving skin and bone and tend to be multifocal. Herein we report a case of LC neoplasm involving the lymph nodes and illustrate the inherent diagnostic pitfalls and pearls.

## CASE REPORT

A 29-year-old male presented with progressively increasing right inguinal, cervical, and left axillary swellings over two and a half months, which measured 20 × 10 cm, 10 × 5 cm, and 7 × 5 cm respectively on clinical examination, along with mild fever. There was no history of night sweats or weight loss. He had consumed homeopathic therapy for 10 days, followed by therapy for tuberculosis for the last 1.5 months. On examination, the liver and spleen were non-palpable. Positron emission tomography (PET) scan revealed an FDG avid (SUV max 23.97) conglomerate nodal mass in the (i) left cervical regions (level II-V), (ii) left axillary region, (iii) retroperitoneal nodal mass extending from the pelvic region to the right external iliac region, and (iv) bilateral inguinal nodes; measuring up to 7.7 × 5.5 cm. Erosion of the left third rib and left pleural infiltration were also identified. The working diagnosis was lymphoma.

**Figure 1:**
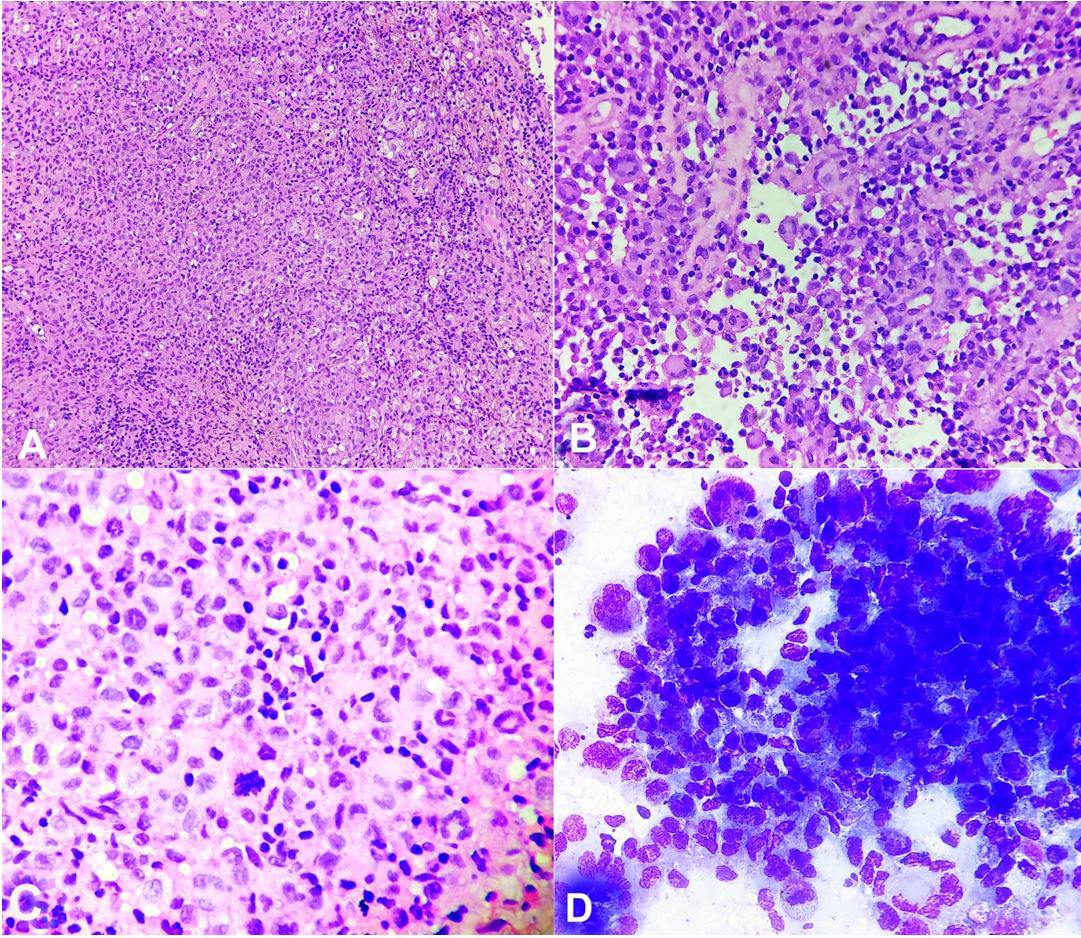
Photomicrograph of the histopathology [H & E, (**A**) 100X, (**B**) & (**C**) 400X] and the cytopathology [Giemsa (**D**) 400X], displaying a few cohesive clusters of medium to large-sized pleomorphic atypical cells with variable morphology admixed with small lymphocytes, histiocytes, few plasma cells, neutrophils, occasional eosinophils, and foamy macrophages. A fair number of the cells were large-sized with wreath-like nuclei and plasmacytoid appearance along with the presence of atypical mitosis.

The cervical lymph node’s core biopsy histopathologic examination (HPE) showed multiple tissue cores infiltrated by medium to large-sized pleomorphic atypical cells with variable morphology admixed with small lymphocytes, histiocytes, and few plasma cells, neutrophils, occasional eosinophils and foamy macrophages. The cells demonstrated moderate atypia and ovoid folded to indented nuclei, fine chromatin, inconspicuous nucleoli and moderate cytoplasm. A fair number of the cells were large-sized with wreath-like nuclei and plasmacytoid appearance (Figures 1A, 1B, and 1C). Mitotic index was high (approximately 10-12/10HPF), including atypical mitosis. The differential diagnosis initially considered on HPE included anaplastic large cell lymphoma, diffuse large B cell lymphoma, metastatic carcinoma, myeloid sarcoma and blastic plasmacytoid dendritic cell neoplasm (BPDCN). On immunohistochemistry, the atypical cells were positive for LCA (weak) and negative for PanCK (AE1/AE3), CD20, CD3, CD5, CD8, CD30, ALK-1, CD43, CD2, CD7, CD68, SOX10 and PAX-5. CD4 staining in the atypical cells was diffuse and of strong intensity. At the same time, Ki-67 proliferative index revealed heterogeneous staining, being approximately 65-70% in the highest proliferative zone. LCA stained the remnant lymphocytes, CD20 and CD3 the B and T-lymphocytes among them, respectively, and CD68 the histiocytes. An unequivocal diagnosis could not be rendered at this moment and the possibilities proffered were BPDCN and myeloid sarcoma. The same was communicated to the attending clinician, and the case was discussed in the multidisciplinary tumor board. The moot point was the lack of handy markers such as CD123 and MPO (myeloperoxidase) on IHC, but fortunately, it was available on flow cytometry. A decision was taken to attempt flow cytometry on the material aspirated by fine-needle cytology from the suspicious lymph nodes.

Thus, fine needle aspiration cytology was performed from the left cervical lymph node mass, and smears were also prepared while subjecting the material to flow cytometry. The cellular smears revealed singly scattered and a few cohesive clusters of pleomorphic neoplastic cells, which were medium to large-sized, with a round to oval irregularly folded subcentric to eccentric vesicular nuclei and moderate to abundant cytoplasm, vacuolated at places. Prominent nuclear grooves were discerned in some of the cells. Many multinucleated giant cells containing wreath-like nuclei were evident, accompanied by increased phagocytic activity and marked cytologic atypia, especially in the cells seen in the clusters (Figure 1D). Cytologic diagnosis suggested neoplasm of histiocytic origin.

**Figure 2:**
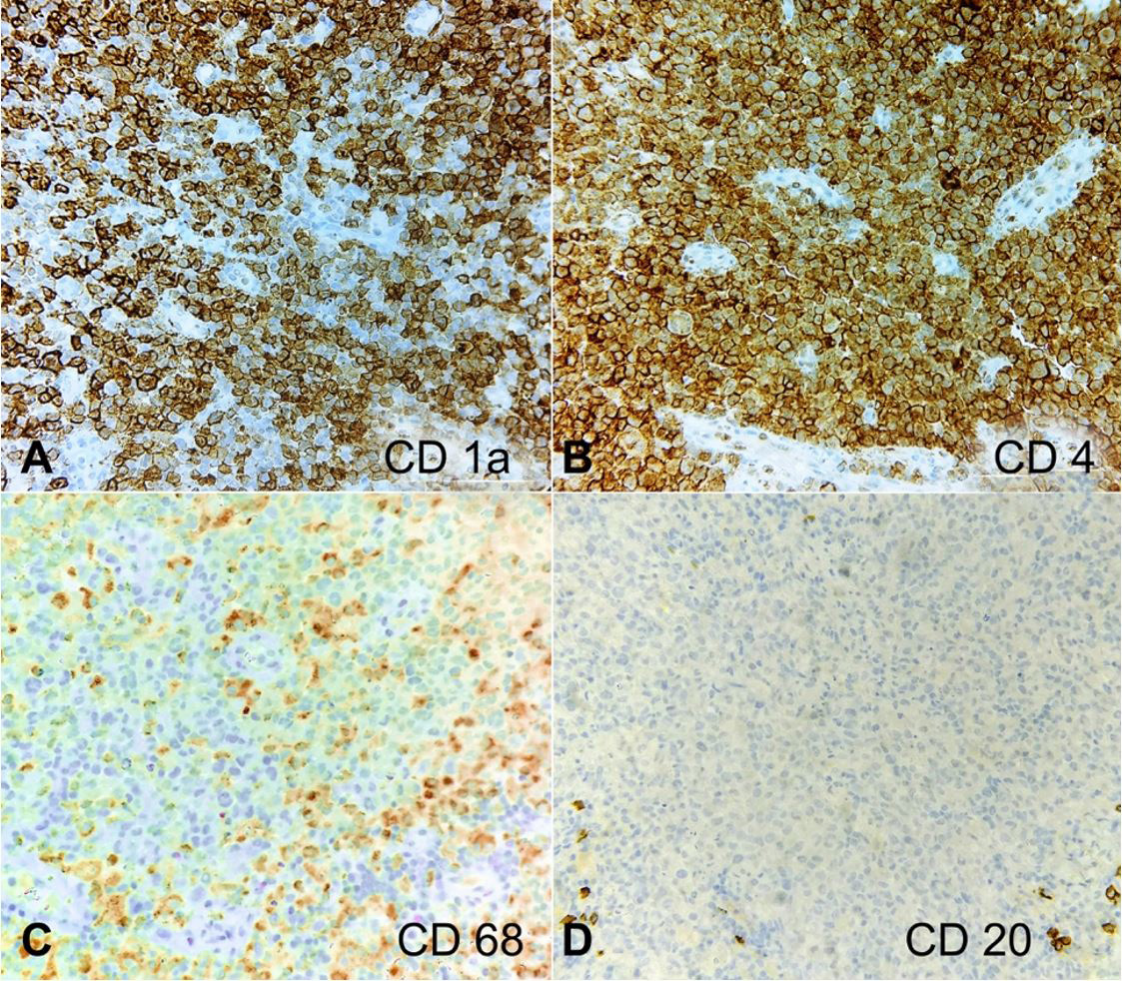
Photomicrograph of the IHC analysis revealing the tumor cells to be positive for CD1a (**A**, 400X), CD4 (**B**, 400X), CD68 [variably] (**C**, 200X) and negative for CD20 (**D**, 200X).

**Figure 3:**
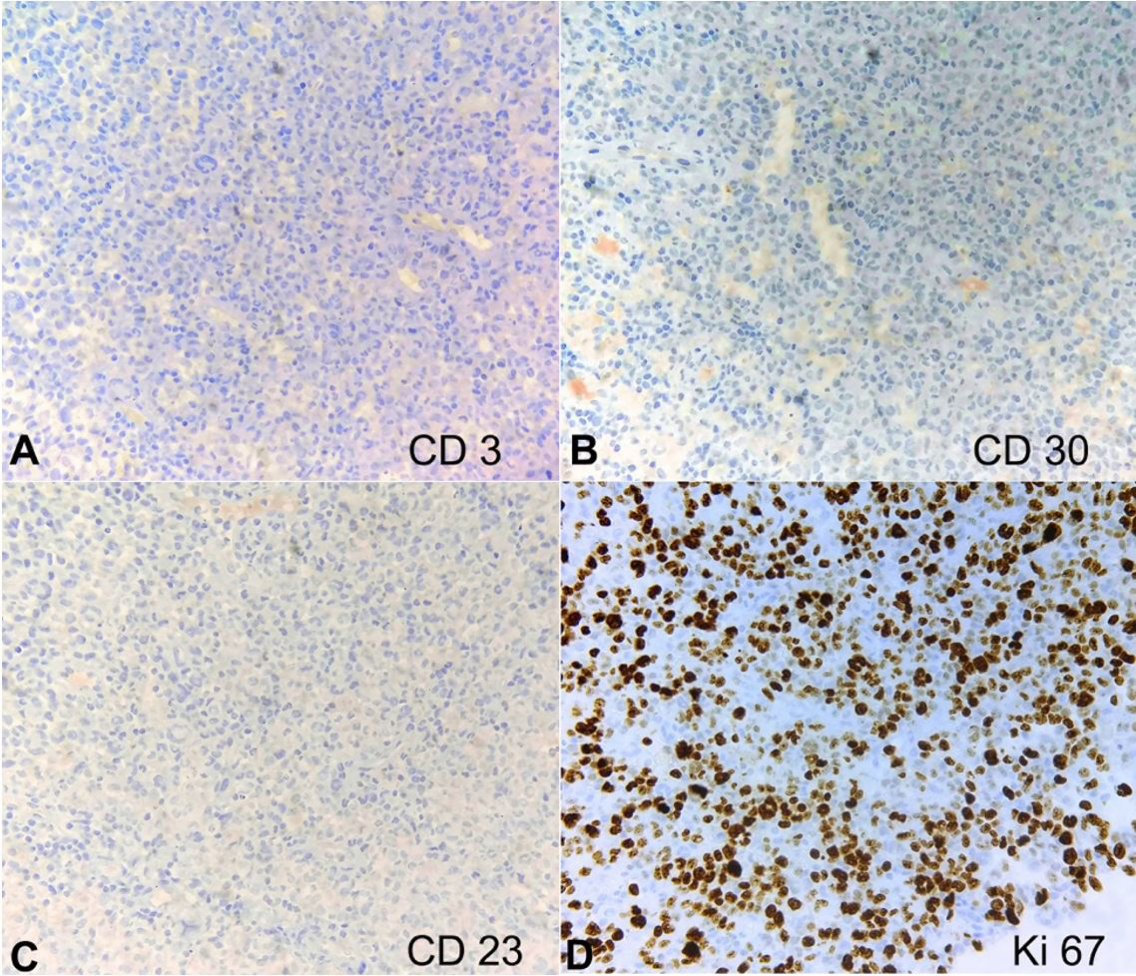
Photomicrograph of the IHC analysis revealing the tumor cells to be negative CD3 (**A**, 200X), CD30 (**B**, 200X), and CD23 (**C**, 200X). Ki67 index (**D**, 400X) was approximately 65-70% in the highest proliferating zone.

**Figure 4:**
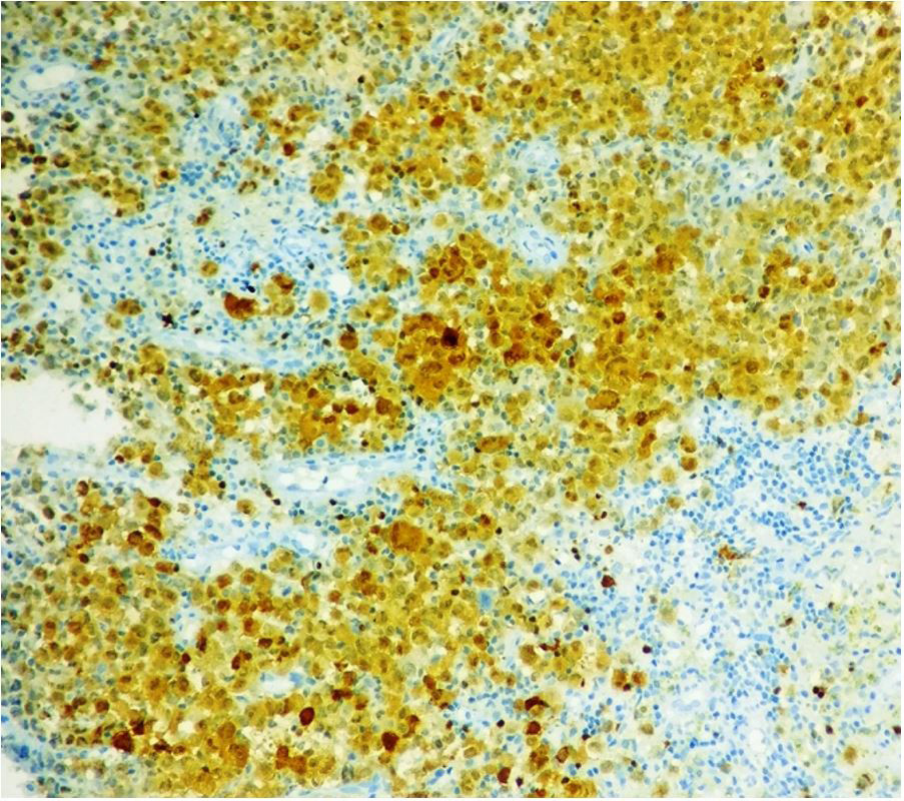
Photomicrograph of the IHC analysis revealing the tumor cells to be positive for S100 (400X).

The histopathology slides were reviewed along with the IHC analysis. Careful and meticulous appraisal evinced nuclear grooves in the atypical cells and variable positivity of CD68 in the atypical cells, thus prompting the consideration of a histiocytic neoplasm, probably of Langerhans cell origin. Additional IHC was resorted to, which illustrated diffuse and strong positivity of the atypical cells for CD1a, S100, and langerin (CD207) and negativity for CD21 and CD23, thus confirming a Langerhans cell neoplasm (Figure 2, 3, and 4). Providing succor was also the flowcytometric finding of negativity of the atypical cells for CD123 and MPO.

However, the dilemma flummoxing the pathologist now was whether the lesion was a Langerhans cell sarcoma or Langerhans cell histiocytosis. In conjunction with the cytopathologic observations and in view of the moderate atypia, high mitotic, and proliferative indices, a final diagnosis of Langerhans cell histiocytosis progressing to Langerhans cell sarcoma was rendered. Molecular analysis, especially for BRAF V600E mutation, was not feasible. Chemotherapy was instituted, and the patient was treated with a combined regimen comprising ifosfamide, carboplatin, and etoposide. After 3 cycles of chemotherapy, the PET-CT scan showed decreased metabolic activity of the retroperitoneal and right inguinofemoral lymph nodes; however, new lesions were detected in the left pleural and right thigh region. Biopsy of the pleural lesion was performed, HPE of which revealed similar histopathologic features as mentioned above and IHC positivity for CD1a, thus reiterating the diagnosis of LCS. The chemotherapy was continued and, until the last follow-up, after having completed 6 cycles of the chemotherapy regimen, a significant reduction in disease activity has been documented.

## DISCUSSION

Langerhans cells were first described by Paul Langerhans in 1868.^3^ Tumors derived from Langerhans cells are classified by the World Health Organization (WHO) into Langerhans cell histiocytosis (LCH) and Langerhans cell sarcoma (LCS). This distinction is based on the degree of cytologic atypia and clinical aggressiveness However, there are rare examples where the distinction and delineation are challenging, probably due to the biologic spectrum of LC neoplasms. LCH is a clinically benign disease; however, rarely it can transform into LCS. LCS is a rare malignant tumor of Langerhans cells displaying features typical of malignant tumors, i.e., rapid growth, local invasion, the ability to recur and metastasize. The incidence of LCH is estimated at five per million per year, whereas that of LCS is unknown.^4^ Almost all reported cases of LCS are in adults. The median patient age is 41 years (range: 10-72 years), and a male:female ratio of 1.4:1.^2^ The skin and underlying soft tissue are the most common sites of involvement. Multiorgan involvement can affect the lymph nodes, lung, liver, spleen, and bones. Most cases are extranodal (involving skin and bone) and multifocal. Only 22% of cases are primarily nodal. Hepatosplenomegaly is noted in 22% and pancytopenia in 11%.^2^ Several etiological factors have been proposed for LCS including immunosuppression, viruses’ infection and prior hematological diseases. Immunosuppression has been linked to increased rates of malignancy (2.7 to 13.7-fold increase post-transplant), with the risk increasing with the intensity and duration of the immunosuppression and with certain immunosuppressive agents e.g., calcineurin inhibitors.^5^

Typically, in LCH, the proliferating cells have characteristic cytologic features of LCs. They are ovoid or elliptic in shape with grooved, folded, indented, or lobulated nuclei having inconspicuous nucleoli and fairly abundant, weakly eosinophilic cytoplasm. Mitotic activity is variable, but atypical mitosis is primarily absent. Associated features in the background include an increase in eosinophils, neutrophils, and histiocytes.^4^ Unlike LCH, the characteristic nuclear features of Langerhans cells are seldom identified in LCS. LCS is a poorly defined high-grade malignant neoplasm composed of large cells with grooved nuclei, granular chromatin, prominent nucleoli, and high mitotic rate (usually > 50/10 HPF). Although inflammatory infiltrate can be abundant, especially if there is associated necrosis, eosinophils are usually scattered and scarce. There appears to be no clear difference in phenotypic profiles between LCH and LCS, expressing the typical immunophenotypic profile of Langerin (CD207), CD1a, S-100 protein, and similar ultra-structure characteristics of Birbeck granules within the cytoplasm.^2^^,^^4^^,^^6^^,^^7^ Although not pathognomonic, the disease is found to harbor BRAF V600E mutation.

The diagnostic conundrums related to tumors derived from Langerhans cells such as the one in the present context can be two-fold: first differentiating the lower grade lesions from other entities such as reactive (non-neoplastic) histiocytic lesions, granulomatous inflammation and LC hyperplasia; and the higher grade lesions from epithelial or mesenchymal malignancies, other histiocytic neoplasms and non-histiocytic hematolymphoid neoplasms such as anaplastic large cell lymphoma, myeloid sarcoma, and BPDCN. The present case exhibited marked atypical proliferation of the lesional cells with significant mitotic activity endorsing it as a neoplasm, rather than hyperplasia or reactive proliferation. Thus, it could be distinguished from the differential diagnoses of the lower grade lesions. Immunohistochemical antibodies, including epithelial, mesenchymal, lymphoid, histiocytic and dendritic cell markers, enabled the delineation of this neoplasm from other high-grade neoplasms as mentioned above. Diagnostic considerations such as anaplastic large cell lymphoma, metastatic carcinoma, and other histiocytic neoplasms were excluded by the negativity of the tumor cells for antibodies such as CD30, ALK, PanCK, CD21, and CD23. Interesting to note was the diffuse positivity of the tumor cells for CD4 with strong intensity. Though CD4 expression is well known in the histiocytic lineage, it was the robust manifestation that popped up as a diagnostic pitfall, leading to the contemplation of BPDCN and myeloid sarcoma. The diagnostic predicament was resolved by the negativity of the tumor cells for markers such as CD123 and MPO and enunciation for CD1a. However, the quandary which posed the most excruciating was the precise characterization of the LC neoplasm – as LCH or LCS. The cytopathology of this case displayed distinct aggregates of cells with unequivocal moderate to marked atypia at places and presence of atypical mitosis. However, definite features of marked atypia and overtly high-grade malignant features such as those of pleomorphic sarcoma evident in cases of LCS were not discerned in this case on histopathology. Considering all of the above, the high Ki67 proliferation index of around 70%, the clinical presentation of rapid spread of the disease, and the high FDG uptake on PET-CT, the final opinion of LCH progressing into an LCS was proffered. Similarly, other authors noted that the differential diagnosis of LCS from LCH may often be challenging due to their histological and immunohistochemical similarities. The discriminating attributes as accounted for in the literature and accepted by the WHO are a markedly higher degree of cytological atypia, more frequent mitotic features and a higher rate of Ki67 proliferation index (usually more than 30%) in LCS compared with LCH.^2^^,^^4^^,^^6^^,^^8^ The tumor in our study did not exhibit high cellularity, cellular crowding, marked pleomorphism and atypia neither in the primary nor in the recurrent pleural lesion, as is the wont of LCS. Thus, despite a relatively rapid recurrence, the neoplasm could not be categorized as unequivocal LCS. The most appropriate terminology in the context of the case considering all the relevant features, after due diligence and insight seemed to be LCH progressing to LCS.

The successful treatment of advanced LCS with multiple organ involvement is feasible with a variety of chemotherapeutic regimens. Systemic combination chemotherapy, such as the CHOP or CHOP-like regimens, may be helpful in some cases.^5^^,^^9^^-^12 Yoshimi et al.^13^ used the ESHAP (etoposide, carboplatin, cytarabine, methylprednisolone) regimen to treat a case refractory to the CHOP regimen, and observed a remarkable improvement. A few others have reported the efficacy of MAID (mesna, doxorubicin, ifosfamide, dacarbazine) regimen in a proportion of patients. Radiotherapy has also been reported to be effective in certain cases.^5^^,^^6^^,^^14^^-^17 Complete remission, without signs of recurrence or metastasis for 45 months without adjuvant therapy, was achieved by a total dose of 59.4 Gy radiotherapy to a cervical lymph node LCS patient.^6^^,^^18^ From the patients with available follow-up data, about 50% died of their disease within 2 years after diagnosis, despite conventional combination chemotherapy, surgery, and radiotherapy. Considering the poor outcome and prognosis of LCS, more aggressive and effective standard therapies are urgently entailed, and a careful follow-up plan is mandated.

In summary, LCS is an extremely rare disease, and primary nodal presentation is even rarer. We essayed the clinical manifestation, immunophenotype, and treatment course of a case of LC neoplasm and attempted to underscore the diagnostic perils and pearls coupled with this entity. Due to the low incidence of LCS, it is crucial to recognize this unique neoplasm and differentiate it from other neoplasms and more importantly from LCH. The salient traits on histopathologic evaluation and IHC are indispensable in the veracious identification of this recherche entity.
